# Integrating In Vitro and In Silico Analysis of a Cationic Antimicrobial Peptide Interaction with Model Membranes of Colistin-Resistant *Pseudomonas aeruginosa* Strains

**DOI:** 10.3390/pharmaceutics14061248

**Published:** 2022-06-12

**Authors:** Sandra Patricia Rivera-Sanchez, Iván Darío Ocampo-Ibáñez, Yamil Liscano, Natalia Martínez, Isamar Muñoz, Marcela Manrique-Moreno, Luis Martinez-Martinez, José Oñate-Garzon

**Affiliations:** 1 Research Group of Microbiology, Industry and Environment, Faculty of Basic Sciences, Universidad Santiago of Cali, Cali 760035, Colombia; ivan.ocampo00@usc.edu.co (I.D.O.-I.); natalia.martinez02@usc.edu.co (N.M.); isamar.munoz00@usc.edu.co (I.M.); 2 Transnational Research Group on Infectious Diseases, PhD School of Biomedicine, University of Córdoba, 14071 Córdoba, Spain; 3 Research Group of Comprehensive Health (GISI), Department Faculty of Health, Universidad Santiago de Cali, Cali 760035, Colombia; yamil.liscano00@usc.edu.co; 4 Chemistry Institute, Faculty of Exact and Natural Sciences, University of Antioquia, Medellin 050010, Colombia; marcela.manrique@udea.edu.co; 5 Microbiology Unit, Reina Sofía University Hospital, 14008 Córdoba, Spain; luis.martinez.martinez.sspa@juntadeandalucia.es; 6 Maimonides Institute for Biomedical Research of Córdoba, 14008 Córdoba, Spain; 7 Department of Agricultural Chemistry, Soil Sciencies and Microbiology, University of Córdoba, 14071 Córdoba, Spain; 8 Research Group of Chemistry and Biotechnology, Faculty of Basic Sciences, Universidad Santiago of Cali, Cali 760035, Colombia

**Keywords:** colistin-resistant *Pseudomonas aeruginosa*, cationic antimicrobial peptides, model membranes, membrane–peptide interaction

## Abstract

Bacterial antibiotic resistance is a serious global public health concern. Infections caused by colistin-resistant *Pseudomonas aeruginosa* (CRPa) strains represent a serious threat due to their considerable morbidity and mortality rates, since most of the current empirical antibiotic therapies are ineffective against these strains. Accordingly, cationic antimicrobial peptides (CAMPs) have emerged as promising alternatives to control resistant bacteria. In this study, the interaction of a CAMP derived from cecropin D-like (∆M2) with model membranes mimicking bacterial biomembranes of wild-type (WTPa) strains of *P. aeruginosa* and CRPa was evaluated through in vitro and in silico approaches. In vitro interaction was determined by infrared spectroscopy, whereas in silico molecular dynamics was performed to predict specific interactions between amino acids of ∆M2 and lipids of model membrane systems. Experimental analysis showed this peptide interacted with the lipids of bacterial-like model membranes of WTPa and CRPa. In both cases, an increase in the concentration of peptides induced an increase in the phase transition temperature of the lipid systems. On the other hand, the peptides in solution underwent a transition from a random to a helical secondary structure after interacting with the membranes mostly favored in the CRPa system. The α-helix structure percentage for ΔM2 interacting with WTPa and CRPa lipid systems was 6.4 and 33.2%, respectively. Finally, molecular dynamics showed ∆M2 to have the most affinities toward the phospholipids palmitoyl-oleyl-phosphatidylglycerol (POPG) and palmitoyl-oleoyl-phosphatidylethanolamine (POPE) that mimic membranes of WTPa and CRPa, respectively. This work provides clues for elucidating the membrane-associated mechanism of action of ∆M2 against colistin-susceptible and -resistant strains of *Pseudomonas aeruginosa.*

## 1. Introduction

Antimicrobial resistance in bacterial pathogens is a worldwide problem that causes significant morbidity and mortality [1,2]. Gram-negative bacteria may be resistant to multiple medicines, such as fluoroquinolones, beta-lactams (including carbapenems), aminoglycosides, polymyxins, and other antibiotics. Antibodies, probiotics, bacteriophages, vaccines, immune stimulation, and antimicrobial peptides are all options for treating multiresistant bacterial infections [3]. According to a report from the United Kingdom, global antimicrobial resistance will cause death to 10 million people by 2050 [2], costing the global economy up to $100 billion [4]. As a result of this issue, the World Health Organization (WHO) has published a list of priority pathogens resistant to antibiotics for which new antibiotics are urgently needed, which includes the 12 families of bacteria with the greatest impact on disease. With priority given to human health, *Acinetobacter baumannii*, Enterobacteriaceae, and *Pseudomonas aeruginosa* are the most dangerous pathogens [2]. *Pseudomonas aeruginosa* is a bacterium commonly found in nosocomial infections that has intrinsic or acquired resistance to a variety of antibiotics, including aminoglycosides, quinolones, and beta-lactams, reducing the number of therapeutic options, tripling mortality in intensive therapy patients, and doubling hospital stays [2,5,6]. In fact, the recent emergence of colistin-resistant *Pseudomonas* strains (CRPa) is a major public health concern around the world, because polymyxins are antimicrobials that are frequently used as a last resort to treat multiresistant bacterial infections [5].Therefore, said resistance involves the decrease or elimination of the early interaction of lipid A based on the charge with colistin. Membrane modifications are produced by the addition of cationic phosphoethanolamine (pEtN) or 4-amino-L-arabinose in lipid A and which result in a decrease in the negative charge on the bacterial surface, slowing down the electrostatic interaction between colistin and the lipopolysaccharide (LPS). These modifications on the bacterial surface are due to overexpression of genes of the chromosome-mediated two-component system (PmrAB and PhoPQ) and mutation in the lipid A biosynthesis genes, causing the bacteria to lose the ability to produce lipid A [3]. Despite peptidic antibiotic resistance, CAMPs are still a viable option for dealing with bacterial resistance. CAMPs are defined as innate immune system effector molecules composed of amino acids ranging from 12 to 60 [7], capable of forming a large number of combinations and, consequently, various sequences to evade CRPa strain resistance.

The NH_3_^+^ groups of the side chains of cationic residues of the CAMP_S_ first interact with the anionic phosphate groups of the lipopolysaccharide (LPS) lipid A component. In addition, hydrophilic residues located on the polar face of the helix contribute to the interaction of hydrogen bonds with sugar moieties of the LPS. Thus, plausible disruption or fluidization of LPS structures facilitate traversal of the peptide through the outer membrane [8], and, subsequently, reaching the inner membrane, alter its integrity [9]. Bacterial membranes are primarily composed of phosphatidylethanolamine (POPE), phosphatidylglycerol (POPG), and cardiolipin (CL) [6,10], and changes in their composition are linked to resistance to CAMPs [11]. For example, it was demonstrated that the CRPa strain has less composition of POPE compared to WTPa, a condition related to polymyxin resistance [12]. However, the role of the bilayer configuration in CAMP resistance is still unknown at the molecular level. Likewise, it was reported that the percentage of lipids in susceptible *P. aeruginosa* is 60% for POPE, 11% for cardiolipin, and 21% for POPG [13]. Whereas in an isolate of *P. aeruginosa* with resistance to polymyxin, the percentage is 5.7% POPG, 18.2% POPE, and 27% CL [14], indicating that the aforementioned results for *P. aeruginosa* with resistance to polymyxin decreased POPE lipids by 26.7% and POPG by 28.6%, and increased CL by 21.1%.

Because of the complexity, structure, and functionality of real membranes, as well as various lipid–lipid and lipid–protein interactions, artificial membrane models are used to study the effect of peptides on membranes [15]. The application of biophysical techniques such as infrared spectroscopy (FT-IR) to study changes in the vibrations of functional groups of phospholipids in a membrane model as a result of peptide interaction was employed in other studies [16,17]. Molecular dynamics (DM) simulation, a bioinformatic tool, can predict the interaction between peptides and membrane models because it is a deterministic technique for visualizing the behavior or progress of a system (physical, chemical, or biological) over time, as it calculates the forces between the atoms that make up the system using various algorithms [18]. 

Cecropin D is a CAMP isolated from *Galleria mellonella* [16,18], which is used as a model for structure–activity relationship studies due to its neutral charge and well-defined hydrophobic/hydrophilic sides of the helix. In a previous study, the ΔM2 peptide was designed by systematic substitutions of anionic/neutral residues for cationic residues on the WT peptide hydrophilic face of the helix, considering the suggestions proposed by [19]. Thus the net charge was increased from 0 to +9. The antibacterial activity was increased about 40-fold in Gram-negative bacteria compared to the neutral WT peptide. Likewise, it was reported that the cationic peptide had a greater permeabilizing effect in Gram-negative bacterial membrane models compared to the neutral [20]. 

Similarly, Rivera et al. 2020 [21] described the antibacterial activity of the ΔM2 peptide against clinical isolates of WTPa and CRPa strains, gaining MIC values of 8 μg/mL and 8–16 μg/mL, respectively [21]. Despite these promising results, the interaction of the ∆M2 peptide on both membrane models is poorly understood. Therefore, the objective of this research was to study the interaction of the ∆M2 peptide with membrane models of susceptible (WTPa) and resistant (CRPa) *P. aeruginosa* from the displacement of the methylene bands with νsCH2 and of the carbonyl νC=O as a function of temperature, by means of in vitro infrared spectroscopy. On the other hand, to understand the effect of the peptide on in vitro membrane models, molecular dynamics (MD) simulations were performed to predict the molecular interaction in both lipid systems. It is expected that this research work constitutes a contribution to the design, development, and obtaining of innovative molecules based on PAMs, that allows the development of new antibacterial drugs.

## 2. Materials and Methods

### 2.1. Peptide Synthesis

The cationic peptide ∆M2 sequence (RNFFKRIRRAGKRIRKAIISAAPAVETLAQAQKIIKGGD), 39-mer, was based on the sequence of a neutral peptide cecropin D from *Galleria mellonella* [22], whose net charge was increased to +9 by means of rational design. The average hydrophobicity of peptide <H> was established using a standard scale [23] and was calculated using the online software Heliquest (http://heliquest.ipmc.cnrs.fr/ accessed on 12 June 2020). The synthesis service of GenScript Corporation (Piscataway, NJ, USA) was requested to obtain the synthetic peptide, which used an automated synthesizer for solid-phase peptide synthesis (SPFS) with 95 percent purity. The lyophilized peptide was dissolved in phosphate buffered saline (PBS). The tool used to find the physicochemical parameters (net charge and hydrophobicity, among others) was Pepcalculator (PepCalc.com—peptide property calculator) (https://pepcalc.com/ accessed on 10 August 2020) [24], which is an online server that consists of an Innovagen peptide calculator where calculations and estimates are made on physicochemical properties.

### 2.2. Representative Synthetic Models from P. aeruginosa Membranes

Based on the membrane composition of WTPa and CRPa bacteria, main lipid components were selected to obtain the synthetic lipid systems used to phase transition and secondary structure analysis. The WTPa membrane is mainly composed of phosphatidylethanoam-ine, phosphatidylglycerol and cardiolipin (POPE: POPG: CL; 65:23:12) [13]. However, in the case of CRPa the proportions change to 35:12:53 respectively [14]. The synthet-ic lipids dimyristoylphosphatidylethanolamine (DMPE, Lot. 160-181PE-139), dimyristoylphosphatidylglycerol (DMPG, Lot. 140PG-167) and cardiolipin (CL, Lot. 750332P-200MG-A-030) were purchased from Avanti Polar Lipids (Alabaster, AL, USA). These lipids were selected because their main transition temperatures are in the BioATR II temperature range.

### 2.3. Supported Lipid Bilayers and Phase Transition Measurements

Supported lipid bilayers of both model membranes were prepared in situ on a BioATR II cell. The unit was integrated to a Tensor II spectrometer (Bruker Optics, Ettlingen, Germany) with a liquid nitrogen MCT detector using a spectral resolution of 4 cm^−1^ and 120 scans per spectrum. The desired temperature was set by a computer-controlled circulating water bath, the Huber Ministat 125 (Huber, Offenburg, Germany). First, the background was taken using 20 mM HEPES buffer, 500 mM NaCl, and 1 mM EDTA in the same temperature range. Subsequently, for coating the silicon crystal, stock solutions of the lipids were dissolved in chloroform. The cell was filled with 20 µL of a 20 mM lipid stock solution, and the chloroform was evaporated resulting in a lipid multilayer film. For in situ measurements, the cell was afterwards filled with 20 µL of buffer or peptide solution at different molar ratios and incubated over the phase transition temperature for 10 min. To determine the position of the vibrational band in the range of the second derivative of the spectra, all the absorbance spectra were cut in the 2970–2820 cm^−1^ range and shifted to a zero baseline, and finally applied the peak-picking function included in OPUS software. The results were plotted as a function of the temperature. To determine the transition temperature (T_m_) of the lipids, the curve was fitted according to the Boltzmann model to calculate the inflection point of the obtained thermal transition curves using the OriginPro 8.0 software (OriginLab Corporation, Northhampton, MA, USA).

### 2.4. Secondary Structure Analysis

Peptide solution was prepared at 1 mM concentration in HEPES buffer. Representative liposomes at a concentration of 5 mM from colistin-susceptible (WTPa) and -resistant *P. aeruginosa* (CRPa) were prepared in buffer according to the proportions described above Section 2.4. For the determination of the secondary structure, peptide and lipid were mixed at the highest concentration evaluated in the phase transition measurements (10 molar%). The experiments were performed at 37 °C in an AquaSpec Cell (Bruker Optics, Ettlingen, Germany). The predictions of secondary structure elements α-helix and β-sheet were performed with the methods supplied by the Confocheck^TM^ system (Bruker Optics, Ettlingen, Germany). These methods calculate the secondary structure with a multivariate partial least squares algorithm (PLS) based on a calibration data set of 45 different proteins.

### 2.5. In Silico Peptide–Membrane Preparation

#### 2.5.1. Structural Modeling and In Silico Validation of Antimicrobial Peptides

Structural modeling of the peptides was achieved using the PEP-FOLD 3 platform (PEP-FOLD 3 version 2.0) (https://bioserv.rpbs.univ-paris-diderot.fr/services/PEP-FOLD3/, accessed on 10 August 2020). Their stereochemical quality was confirmed using Ramachandran plots in MOLPROBITY (http://molprobity.biochem.duke.edu/ accessed on 10 August 2020) [25]. All structures were visualized with PyMOL (version 2.5.0) (https://pymol.org/2/, accessed on 10 August 2020) [26] and Discovery (version 2020) (http://accelrys.com, accessed on 10 August 2020) [27]. 

#### 2.5.2. In Silico Construction of a *Pseudomonas aeruginosa* Membrane Model

CHARMM-GUI (https://www.charmm-gui.org/), accessed on 8 July 2021 was used to create the *P. aeruginosa* membrane model in silico. It is a web platform that is ideal for building complex systems interactively and preparing their inputs with protocols. Well-established and reproducible simulation tools are used for cutting-edge molecular simulations, such as CHARMM, NAMD, and GROMACS [28]. To be able to construct a membrane that met all of the required parameters for this work, the construction of the *P. aeruginosa* membrane model was continued using 3 types of phospholipids, including 1-palmitoyl-2-oleoyl-sn-glycerol-3-phosphatidylethanolamine (POPE), cardiolipin (CL), and 1-palmitoyl-2-oleoyl-sn-glycero-3-(phospho-rac-(1-glycerol)) (POPG). The number of molecules in the outer and inner monolayers of the in silico membrane model of WTPa was 65% POPE, 12% CL, and 23% POPG [13] and for CRPa it was 35% POPE, 53% CL, and 12 POPG.

### 2.6. Molecular Dynamics and Analysis

For the molecular dynamics simulation of the peptide, version 2019.3 of the GROMACS software (GROMACS 2020) was used. In the ion placement method, 0.5 M NaCl and a water thickness of 22.5 Å were used with CHARMM36m (https://www.charmm-gui.org/) accessed on 8 July 2021 as a force field, which is appropriate for describing the distribution of molecules within a large system, such as membranes. The systems were calibrated by gradually heating to 310.15 K at 1 femtosecond (fs)/step for 75 picoseconds (ps), in order to certify that the system did not exhibit steric clashes or inappropriate geometry, and at 2 fs/step for 300 ps in the balanced step.

The energy of the system was minimized using the descent algorithm in 5000 steps with a tolerance value of 1000 kJ mol^−1^ nm^−1^ and the Verlet cutoff scheme. Berendsen’s algorithm with 125,000 n steps was used to equilibrate the system temperature and pressure. To balance the system, the Nosé–Hoover and Parrinello–Rahman algorithms were used to adjust temperature and pressure for 50 nanoseconds (ns) during data collection. To account for long-range electrostatic interactions, particle mesh Ewald (PME) summation was used [29]. 

Molecular dynamics (DM) was used to collect data for 50 ns because this is enough time to determine whether or not the peptide contacted the membrane. To account for long-range electrostatic interactions, particle mesh Ewald (PME) summation was used. The SHAKE algorithm constrained the bonds containing hydrogen atoms to their lowest energy values, allowing a numerical integration time interval of 2 fs to be used in the simulation [29].

The interaction of the ΔM2 peptide with phospholipids was studied using GROMACS version 2020 (https://manual.gromacs.org/documentation/, accessed on 8 July 2021) [28,29] to better understand its mechanism of action. The root-mean-square deviation (RMSD) was used to accomplish this. Intermolecular interactions between peptide and membrane phospholipids were studied to see if there were any differences between the membranes of susceptible and resistant Pseudomonas. Using tools such as PYMOL (version 2.5.0) (https://pymol.org/, accessed on 8 July 2021) [26], and Discovery Studio Visualizer (2020) (http://accelrys.com, accessed on 8 July 2021) [27], sampling was performed every 5 nanoseconds until 50 ns, obtaining the residues with the most interactions and the peptide with affinity for certain phospholipids in each type of membrane.

## 3. Results and Discusión

### 3.1. Physicochemical Parameters

Antimicrobial peptides have various physicochemical and structural characteristics, which must be taken into account because they play a fundamental role in the regulation of their antimicrobial activity, their mechanism of action, and their toxic properties [30]. The ∆M2 peptide has a cationic charge of +9, a hydrophobicity of 46.3%, and a length of 39 amino acids. This charge is a positive characteristic for predicting some type of interaction between the cationic charge of the peptide and the anionic charge present in the membranes of the Gram-negative bacteria of WTPa and CRPa. According to studies described by [31], by substituting residues, such as histidines for lysines, the charge increased from +3 to +5 in the CNY21 peptide, allowing an increase in the permeability of larger and more valent ions to scramble the bacterial membranes [31]. Similarly, the charge is responsible for the initial electrostatic interactions between the peptide and the anionic microbial membrane. Thus, several studies support that increasing the charge on a peptide sequence improves antimicrobial activity [11]. On the other hand, the hydrophobicity found for peptide ∆M2 does not exceed the threshold of 50%, indicating that the peptide does not have high hemolytic activity for mammalian cells and its antimicrobial activity is not diminished [15,30]. 

### 3.2. Phase Transition Measurements 

**Figure 1:**
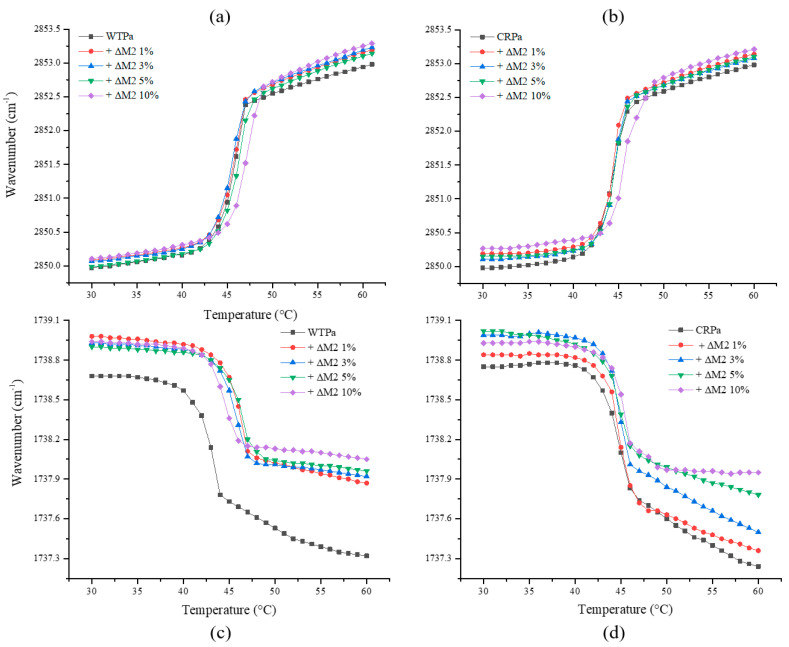
Peak positions of the symmetric stretching vibration bands of the methylene groups and stretching carbonyl vibration measured by FT-IR as a function of temperature. Panels (**a**,**b**) correspond to the ν_s_CH_2_ vibration of *P. aeruginosa* (WTPa) and colistin-resistant *P. aeruginosa* (CRPa), respectively. Panels (**c**,**d**) correspond to the C=O vibrational bands of *P. aeruginosa* (WTPa) and colistin-resistant *P. aeruginosa* (CRPa) in the presence of different concentrations of ΔM2.

To follow the conformational order of the lipid chains in the *P. aeruginosa* model membranes under study, the symmetric CH_2_ vibrational mode (ν_s_CH_2_) was monitored in the IR-spectra in a spectral range of 2970 to 2820 cm^−1^, as indicator for the gel-to-fluid membrane phase transition. The increasing temperature induced changes in the trans-gauche ratio of the acyl chains, and a shift in the maximum wavenumber position of this specific band is considered as a marker for the fluidity and change in phase behavior of the membrane. In the gel phase, ν_s_CH_2_ lies at 2850 cm^−1^, and in the liquid crystalline phase, around 2852 to 2853 cm^−1^ [32,33]; WTPa and CRPa model membranes were prepared to accurately represent the membrane behavior. Figure 1a shows the temperature dependence of the wavenumber values of the peak positions of the POPE:POPG:CL (65:23:12) acyl chains for the WTPa system and for the mixtures at different concentrations of ΔM2. The phase transition of the pure lipid system results in a sigmoidal curve that showed an inflection point that corresponds to the main transition temperature (T_m_) at 45.6 °C. Increasing concentrations of ΔM2 induced a slight fluidization effect, which is more evident above the main transition temperature by the increase in the wavenumbers of the ν_s_CH_2_ at fixed temperatures. The T_m_ was only shifted at the highest concentration evaluated of 10 molar% with a shift upwards of as much as 1.6 °C, suggesting a stabilizing effect on the membrane. The increase in the bilayer order could be due to the shielding of the polar group charges after peptide binding avoiding the lateral repulsion between the lipids and, therefore, the gel phase is stabilized [34]. The results of the interaction of ΔM2 and CRPa model membranes (POPE:POPG:CL 35:12:53) is summarized in Figure 1b. The main transition of the pure lipid system was 44.5 °C. Increasing peptide concentrations induced a higher fluidization effect in comparison with the WTPa lipid system; the effect of the peptide was evident below and above the main transition. However, the Tm of the lipid system was only affected at the highest concentration evaluated (10 molar%), where there was a shift of 1.6 °C. 

Additionally, the hydration of the headgroup region of both lipid systems was characterized by analyzing the carbonyl stretching vibration. The vibrational frequency of the lipid ester C=O groups is sensitive to hydration and, thus, shifted to lower wavenumbers. Figure 1c shows the temperature dependence of the carbonyl stretching vibration of the *WTPa* model membrane. A similar behavior was observed at the peak position of the wavenumber of the gel and liquid-crystalline phase at all the concentrations evaluated of ΔM2, suggesting that the peptide strongly interacted with the phospholipid headgroups. The C=O vibration band depends on the strength of hydrogen bonds established with the water molecules [35]. Peptide–membrane interactions can alter the water dynamics of the polar phospholipid headgroups in the interphase region [36], even at ~10 Å from the membrane interphase plane [37]. Above the main transition temperature, the concentration of 10 molar% induced the highest change in hydration at the interface, suggesting that water molecules are excluded [18,37] as a result of the increased packing of acyclic chains revealed above. However, water molecules may also be solvating side chains of polar amino acid residues, reorienting them more towards the peptide while interactions with C=O are reduced. Interestingly, the analysis of the results of ΔM2 and the carbonyl stretching vibration of the CRPa membrane showed a gradual change in the vibration that is directly related to the concentration of the peptide (Figure 1d). To comprehend these findings, it is necessary to consider the number and type of phospholipids that comprise each membrane. For example, the CRPa-mimicking membrane contains considerable and more cardiolipin than the WTPa membrane, implying that the latter prevents abrupt changes in hydration at the C=O level, particularly in the gel and liquid crystalline phases. Cardiolipin is distinguished by a small alcohol head shared by two phosphatidate groups. When compared to both phosphatidates, the head area is significantly less. This should promote greater cohesion between lateral CL [38], preventing the peptide from causing significant changes in carbonyl hydration at low concentrations. Furthermore, its geometry limits its flexibility and mobility [39], affecting the mobility of the polar head and, as a result, its ability to interact with other molecules. These findings, however, will be discussed in greater detail below.

### 3.3. Secondary Structure Prediction

The analysis of the results showed that the ΔM2 peptide did not present a secondary structure in buffer solution. This result is in accordance with the literature, where it is de-scribed that peptides are usually unfolded in aqueous environments, but generally fold during their interaction with membranes [40]. The prediction of the α-helix structure for ΔM2 and the colistin-susceptible *P. aeruginosa* system showed a conformational change of 6.4% and the prediction of the secondary structure of ΔM2 and the colistin-resistant *P. aeruginosa* system showed a conformational change of 33.2%. When peptides bind to a membrane, they undergo conformational transitions from a random structure to a helical structure, which is advantageous in amphipathic environments such as membranes. [17] The α-helix is one of the most common structures in peptides, with hydrophobic residues on one side of the helix and cationic/hydrophilic residues on the other, and both sides of the helix being important for antibacterial activity [41].

### 3.4. Interaction between Peptides and Membranes 

**Figure 2:**
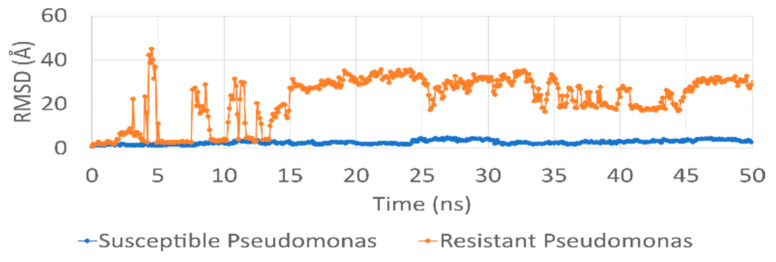
RMSD (root-mean-square deviation) between ΔM2 peptide (one molecule) and *Pseudomonas* membrane models through 50 nanoseconds. Susceptible membrane system model and ΔM2 peptide (blue line). Resistant membrane system model and ΔM2 peptide (red line).

In order to understand the results found from the in vitro interaction of the peptide ΔM2 with the synthetic membrane models of colistin-sensitive *P. aeruginosa* (WTPa) and colistin-resistant *P. aeruginosa* (CRPa), the interaction was explored by molecular dynamics as in previous studies where molecular dynamics models were used to explore the peptide–membrane interactions [23,29]. In Figure 2, the root-mean-square deviation (RMSD) of the ∆M2 peptide is compared with the WTPa and CRPa membrane models, displaying a greater fluctuation of the peptide on the CRPa membrane due to a greater degree of divergence of the aligned structures (by movements) [25]. In WTPa membrane models, it is seen that the system reaches a significant stability from the first moment of the interaction, presenting variations in the RMSD ranging from 2 to 5 Å and suggesting that there is strong association between the peptide and the membrane surface. In the case of the CRPa membrane models, however, a high dynamism of the peptide–membrane system is observed during the first 15 nanoseconds of simulation, reaching oscillations in the RMSD from 10 to 45 Å as a consequence of the initial interactions and of the insertion process of the peptide into the polar phospholipid head group. The system achieves greater stability after 15 ns, indicating that the peptide properly fit within the phospholipid polar head. The type and number of interactions that are established within the system as a function of time must be revealed in order to understand these behaviors.

**Figure 3:**
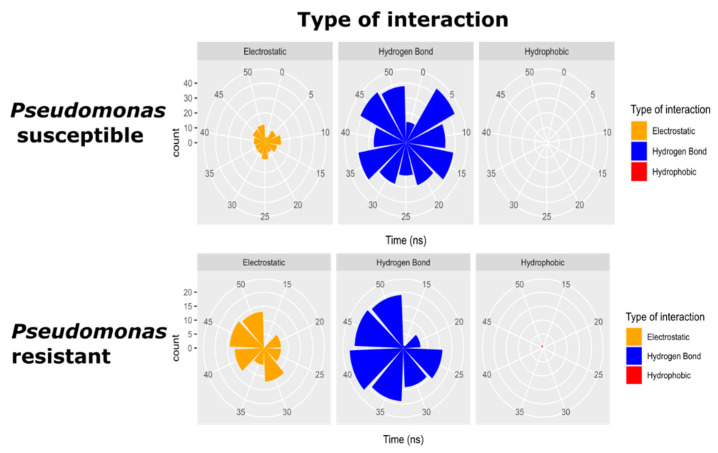
Type of intermolecular interactions between ΔM2 peptide and *Pseudomonas* membrane models through 50 nanoseconds. Electrostatic interaction (yellow). Hydrogen bond interaction (blue) and hydrophobic interaction (red).

**Figure 4:**
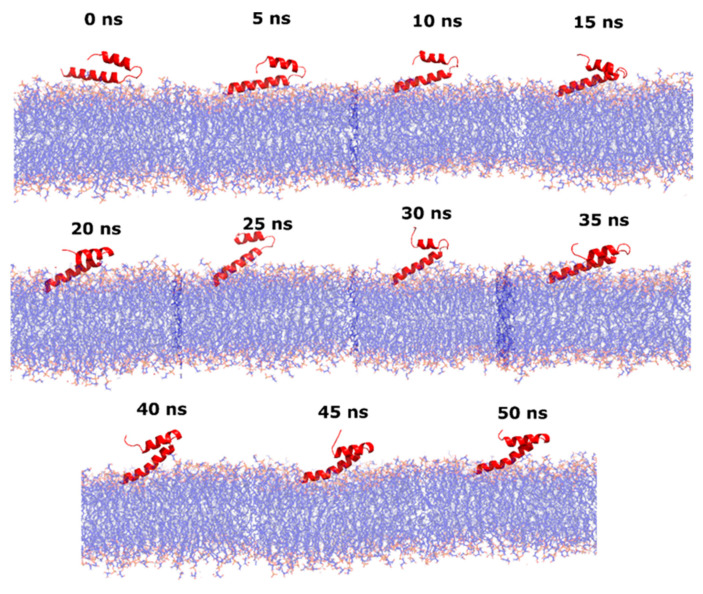
Dynamics of interaction between the ΔM2 peptide and the model membrane of susceptible *Pseudomonas* (WTPa) for 50 nanoseconds.

**Figure 5:**
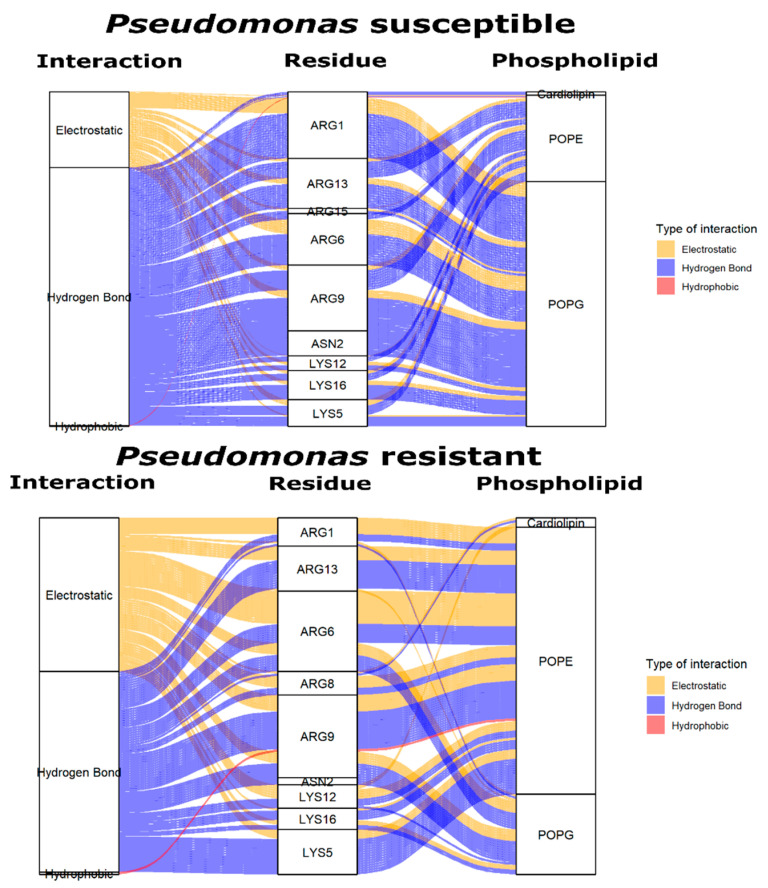
Type of intermolecular interaction with ΔM2 residues and type of phospholipids with more interactions between the peptide and the two types of *Pseudomonas* membrane models (susceptible and resistant). Electrostatic interaction (yellow), hydrogen bond interaction (blue), and hydrophobic interaction (red). Phospholipids POPE (phosphatidylethanolamine), POPG (phosphatidylglycerol).

Subtle fluctuations in WTPa membrane models may be due to the emergence of electrostatic-type interactions and hydrogen bonds (Figure 3) between the peptide and the polar region of the phospholipids from 0 ns. At 25 ns, a difference of 5 Å is detected, possibly due to the fact that the NH-terminal region of the peptide is oriented towards the hydrophobic core of the membrane (Figure 4) since the partitioning of the peptide within the bilayer entails conformational changes facilitated in part by hydrogen bonding [42], reflected in subtle variations of the RMSD. Yet, the peptide manages to insert only up to the interfacial region, in accordance with the increase in Tm and the change in hydration at the carbonyl level, demonstrated in the outcomes obtained by infrared spectroscopy. This strong adherence of the peptide to the surface region is the consequence of multiple interactions, such as hydrogen bonds between POPG and 4 arginines (ARG), 1 asparagine (ASN) and 3 lysines (LYS), in addition to electrostatic interactions between POPE and 2 ARG, CL and 1 ARG, and POPG and 4 ARG and 3 LYS (Figure 5). Similarly, the basic polar residues of arginine and lysine played an important role in the interaction, particularly with POPE. The residues with the most interactions with this phospholipid are ARG6 and ARG9. Thus, the residue that contributes the most to the formation of intermolecular interactions is arginine, which has a greater affinity to bind to the phosphate groups in the bacterial membrane due to the greater number of hydrogen bonds and exactly five donor stabilizer groups [43]. In fact, ARG attracts water molecules in the polar head of the membrane in addition to being electrostatically attracted by phosphate [44], which may be related to the C=O dehydration discussed above. In addition, it was shown that the ARG side chain may approach within 5 Å of the POPG head [45]. 

The likelihood of intermolecular interactions with phospholipids is determined by the number and type of phospholipid. After 5 ns, for example, there were more hydrogen bond interactions than electrostatic ones (Figure 3). The phospholipid POPG is not the principal component of WTPa membranes; however, it has two −OH groups in the polar headgroup and is responsible for most of the H bonds (Figure 5). POPE, the most abundant phospholipid, has an amino group on the polar head that contributes additional linkages, but not as frequently as POPG. This may be due to the fact that the NH_3_^+^ group of POPE binds with oxygen from unesterified phosphate by very close contacts [17], hindering the reorientation of the amino group towards the peptide. On the other hand, the PO_4_^−^ group would be responsible for the electrostatic interactions, more frequently at 10 and 50 ns, in addition to additional H bonds [46].

It was revealed that the membrane model that simulates CRPa has a higher frequency of electrostatic interactions and hydrogen bonds than the susceptible membrane (Figure 3). Electrostatic interactions between CL and 1 ARG, POPE and 4 ARG and 3 LYS, and POPG and 2 ARG and 1 LYS were visualized. On the other hand, hydrogen bonds between POPE and 4 ARG and 3 LYS, and POPG and 2 ARG, 1 ASN, and 2 LYS also were identified. Interestingly, despite the drastic decrease in POPE, this phospholipid had the highest number of interactions as opposed to CL, which increased significantly in molar ratio but not in frequency of interactions. In addition, in contrast to WTPa membrane models, the peptide had a higher affinity for POPE in CRPa systems, suggesting that the polar head of POPE could be involved in the resistance mechanism. Both hydrogen bonds and electrostatic interactions emerged from 20 ns of simulation compared to susceptible membranes appearing from 5 ns. Such results suggest that the presence of the –OH of the POPG polar headgroup in WTPa is indispensable for a fast initial interaction, whereas the interactions with the NH_3_^+^ group of POPE could take 15 ns more, which on a molecular scale could mean variations in the ability to destabilize the membrane, such as differences between the susceptible and resistant membrane models in relation to the hydration of the C=O group (Figure 1c,d).

The geometry of the CL must be considered to understand the peptide selectivity for POPE in the CRPa membrane model. One CL was shown to coordinate two molecules of POPE in a hexagonal lattice [47]. As a result, because the polar head of CL is small in comparison to the phosphatidate groups, the headgroup would not hold the interaction of POPE with the peptide, as might occur with the polar head of POPG in the WTPa membrane model. As a result, the polar headgroup of POPE would be easier to reorient towards the peptide. 

**Figure 6:**
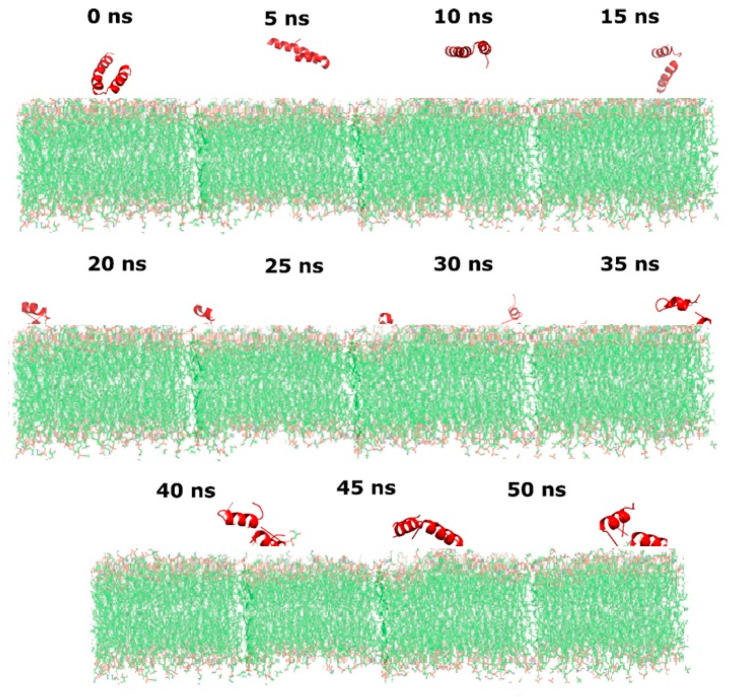
Dynamics of interaction between ΔM2 peptide and colistin-resistant *Pseudomonas* (CRPa) model membrane for 50 nanoseconds.

Finally, the increase in the number of interactions in CRPa membrane models may have biological significance, as these interactions can increase the peptide’s surface anchoring (Figure 6). In this way, the peptide would not have sufficient dynamism in the polar head of the phospholipids to perturb the membrane and generate the death of *P. aeruginosa* in lower concentrations.

## 4. Conclusions 

In this study, an in vitro interaction between peptide ΔM2 with the bacterium-like model membrane lipids of WTPa and CRPa was found, but increasing concentrations of peptide ΔM2 induced a greater fluidization effect in the lipid system of CRPa. Furthermore, it was demonstrated that this interaction is strong for both WTPa and CRPa membrane systems in the polar head of the phospholipids due to the presence of carbonyls (C=O groups) that are sensitive to hydration. Equally, the prediction of the α-helix structure for ΔM2 and the WTPa system displayed a conformational change of 6.4% and the prediction of the secondary structure of ΔM2 and the CRPa system showed a conformational change of 33.2%. These outcomes allow us to explain why there was greater interaction and fluidity in the CRPa membrane systems. It is imperative to note that the α-helix is one of the most usual structures in peptides and is associated to antibacterial activity. Likewise, the DM outcomes revealed and established a high activity of the ΔM2 peptide against WTPa and CRPa membrane models; nonetheless, in the CRPa membrane models, it was evinced that there is a higher frequency of electrostatic interactions compared to in the WTPa membrane, probably due to the presence of CL which directs the peptide to interact with the phosphate group of POPE and to the CL-POPE binding. Despite the fact that 50 ns is a short simulation time, it provided the results required to understand the interaction with the membranes.

Finally, it was demonstrated that the amino acid that interacted the most with the two membrane models of WTPa and CRPa was ARG, which is a critical component of the interaction due to its positive charge. Peptides derived from cecropin D have the potential to inhibit CRPa strains.

## Data Availability

Not applicable.
